# Preventable Tooth Loss in Hawai‘i: The Role of Socioeconomic Status, Diabetes, and Dental Visits

**DOI:** 10.5888/pcd14.170214

**Published:** 2017-11-16

**Authors:** Mikako Deguchi, Marjorie K. Leimomi Mala Mau, James Davis, Richard Niederman

**Affiliations:** 1 Department of Native Hawaiian Health, John A. Burns School of Medicine, University of Hawai‘i, Honolulu, Hawai‘i; 2 Office of Biostatistics and Quantitative Health Sciences, John A. Burns School of Medicine, University of Hawai‘i, Honolulu, Hawai‘i; 3 Department of Epidemiology and Health Promotion, College of Dentistry, New York University, New York, New York

## Abstract

**Introduction:**

Tooth preservation in adults and children is one of the Healthy People 2020 goals for oral health. Although the overall prevalence of tooth loss has been declining in the United States, substantial racial/ethnic differences in preventable tooth loss persist as a public health problem. We examined the strength of the association of health risk factors and tooth loss in Hawai‘i.

**Methods:**

We used data from the Hawai‘i Behavioral Risk Factor Surveillance System survey collected from 2011 through 2014. Participant responses were included if they self-identified as Native Hawaiian, white, Japanese, or Filipino. Differences in excess tooth loss (6 or more teeth) and known risk factors (demographics, diabetes, and dental visits) were analyzed by using univariate analyses and adjusted stepwise, logistic regression models.

**Results:**

We identified oral health inequity among the 4 ethnic groups studied; among the groups, Native Hawaiians had the largest proportion of excess tooth loss. The univariate analyses found differences in the strength of these associations among the 4 racial/ethnic groups. The stepwise analyses found that the associations of excess tooth loss and race/ethnicity were not significant after adjusting for demographics, diabetes status, and dental visits.

**Conclusion:**

Findings suggest a need for programs and policies that improve access to oral health care in Hawai‘i for those with low levels of income and education and those with diabetes.

**MEDSCAPE CME**

Medscape, LLC is pleased to provide online continuing medical education (CME) for this journal article, allowing clinicians the opportunity to earn CME credit.

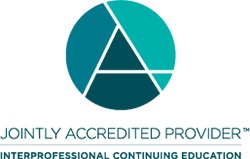
In support of improving patient care, this activity has been planned and implemented by Medscape, LLC and Preventing Chronic Disease. Medscape, LLC is jointly accredited by the Accreditation Council for Continuing Medical Education (ACCME), the Accreditation Council for Pharmacy Education (ACPE), and the American Nurses Credentialing Center (ANCC), to provide continuing education for the healthcare team.

Medscape, LLC designates this Journal-based CME activity for a maximum of 1.00 *AMA PRA Category 1 Credit(s)™*. Physicians should claim only the credit commensurate with the extent of their participation in the activity.

All other clinicians completing this activity will be issued a certificate of participation. To participate in this journal CME activity: (1) review the learning objectives and author disclosures; (2) study the education content; (3) take the post-test with a 75% minimum passing score and complete the evaluation at http://www.medscape.org/journal/pcd; (4) view/print certificate.

**Release date: November 16, 2017; Expiration date: November 16, 2018**

**Learning Objectives**

Upon completion of this activity, participants will be able to:

1. Describe associations of tooth loss in Hawaii with race or ethnicity and other demographic factors, based on an analysis of data from the Hawaii Behavioral Risk Factor Surveillance Survey
2. Distinguish associations of tooth loss in Hawaii with various health and healthcare factors
3. Determine the clinical implications of factors associated with tooth loss in Hawaii

**EDITOR**

Caran Wilbanks

Editor, *Preventing Chronic Disease*

Disclosure: Caran Wilbanks has disclosed no relevant financial relationships.

**CME AUTHOR**

Laurie Barclay, MD

Freelance writer and reviewer, Medscape, LLC

Disclosure: Laurie Barclay, MD, has disclosed the following relevant financial relationships:

Owns stock, stock options, or bonds from: Alnylam; Biogen; Pfizer Inc.

**AUTHORS**

Mikako Deguchi, DDS, MBA

Department of Native Hawaiian Health, John A. Burns School of Medicine, University of Hawaii at Manoa, Honolulu, Hawaii

Disclosure: Mikako Deguchi, DDS, MBA, has disclosed no relevant financial relationships.

Marjorie K. Leimomi Mala Mau, MD, MS

Department of Native Hawaiian Health, John A. Burns School of Medicine, University of Hawaii at Manoa, Honolulu, Hawaii

Disclosure: Marjorie K. Leimomi Mala Mau, MD, MS, has disclosed no relevant financial relationships.

James Davis, PhD

Office of Biostatistics & Quantitative Health Sciences, John A. Burns School of Medicine, University of Hawaii at Manoa, Honolulu, Hawaii

Disclosure: James Davis, PhD, has disclosed no relevant financial relationships.

Richard Niederman, DMD, MS

Department of Epidemiology & Health Promotion, New York University College of Dentistry, New York, New York

Disclosure: Richard Niederman, DMD, MS, has disclosed no relevant financial relationships.

## Introduction

The US Healthy People 2020 includes objective OH-4: reduce the proportion of adults who have ever had a permanent tooth extracted because of dental caries or periodontal disease (1). Healthy People 2020 also sets age-specific goals for adults aged 45 to 64 years (OH-4.1: reduce the proportion of adults aged 45 to 64 years who have ever had a permanent tooth extracted because of dental caries or periodontal disease) and for adults aged 65 to 74 years (OH-4.2, reduce the proportion of adults aged 65 to 74 years who have lost all of their natural teeth). The overall prevalence of tooth loss and edentulism had been declining in the United States from 1972 to 2008, and it has begun to improve (2–4). However, the improvement in dental health has not been shared equally across the United States, with substantial differences among racial/ethnic populations at highest risk (3,5). Tooth loss is a sensitive indicator of overall dental health and access to dental care (6), and oral health in general is correlated with overall health status. Oral health is also associated with other disproportionately poor health outcomes in minority populations, such as diabetes mellitus (7–10), and is often affected by other sociodemographic factors such as unemployment (11). Hence, understanding the relationship of excess tooth loss in the context of other risk factors is critical to reversing poor dental health among high-risk minority populations.

Previous studies have documented associations between tooth loss and demographic status (2,12,13), dental care access (9,14,15), and diabetes (16–19). However, few studies have looked into these factors together and by racial/ethnic groups, particularly among Native Hawaiians and other Pacific Islanders. A first step toward improving oral health among Native Hawaiians and other Pacific Islanders is the identification of racial/ethnic tooth loss prevalence and its relationship to systemic diseases. This study begins to fill this oral health information gap. We examined the differences in the occurrence of tooth loss among whites, Filipinos, Japanese, and Native Hawaiians. Thus, the purpose of this study was to examine the association of excess tooth loss with sociodemographic and health risk factors across 4 racial and ethnic populations in Hawai‘i.

## Methods

Tooth loss and dental visit questionnaires appear on the Behavioral Risk Factor Surveillance System (BRFSS) survey for 2012 and 2014. The survey participants were from Hawai‘i and were interviewed from 2011 through 2014. BRFSS is conducted yearly by the Centers for Disease Control and Prevention to assess a nationally representative sample of noninstitutionalized adults. The study data were provided by the Hawai‘i Department of Health, which collects more detailed information on Hawaiian ethnicities than is available nationally. The study was designed to understand risk factors (demographic and health factors) by ethnicity for having lost 6 or more teeth. Participants were included if they responded to the Hawai‘i BRFSS oral health survey from 2011 through 2014 and self-identified as white, Japanese, Filipino, or Native Hawaiian, restricted to those aged 25 years or older. (Because of the sample size, the 4 ethnic groups were included in this analysis.) The data provided by the Hawai‘i Department of Health did not distinguish Hispanic and non-Hispanic whites. The University of Hawai‘i institutional review board reviewed this study and granted an exemption from review.

Excess tooth loss was defined as removal of 6 or more teeth, based on the persons’ response to a question asking how many permanent teeth have been removed. Answer selections were none, 1 to 5, 6 or more but not all, and all. The answers regarding tooth loss of none and 1 to 5 lost teeth were combined in the analyses. The analyses included both demographic variables (eg, age, income level, marital status, and education) and health characteristics (eg, diabetes, body mass index [BMI]) as possible predictors of having lost 6 or more teeth. Predictors of tooth loss were clustered according to the following: 1) demographic variables — age, sex, race/ethnicity, education, marital status, income level, having health insurance, and residence by metropolitan statistical area; and 2) health characteristic variables — having had a dental visit in the past year, diabetes status (eg, not having diabetes, having prediabetes, and having diabetes), and BMI. BMI (calculated as weight in kilograms divided by height in meters squared [kg/m^2^]) was clinically defined as normal (18.5 to 24.9), overweight (25.0 to 29.9), and obese (≥30). Underweight participants were excluded (n = 827). Race and ethnicity were determined by self-report using a standardized approach for designating multiracial individuals used by the Hawai‘i State Department of Health (20). Having diabetes was self-reported in response to a question asking participants if a doctor had ever told them they had diabetes.

For descriptive analyses, the percentages of demographic and health characteristics are summarized by race/ethnicity. Analyses with having lost more than 6 teeth as the outcome employed logistic regression models. Separate models were initially fit for the 4 ethnicities in the study. Predictor variables initially were analyzed in separate univariate models for the 4 ethnicities in the study. Subsequently, stepwise, multivariable models were fit that included all of the study participants. The stepwise approach was chosen to understand the effects of extending the regression models on the strengths of the associations of the predictors with tooth loss. The first step examined associations of demographic factors with having lost 6 or more teeth. A second step added diabetes, a disease for which there are disparities in prevalence by ethnicity in Hawai‘i, to the model. The final step added having had a dental visit in the past year, to assess the effect of professional dental care. Results of the logistic regression models were reported as odds ratios with 95% confidence intervals. *P* values were determined by Wald tests. All analyses were performed using SAS version 9.4 (SAS Institute, Inc) and included the stratification and weight variables that account for the complex survey design of the BRFSS survey. Significance was set at *P* < .05.

## Results

A total of 13,371 adults were surveyed from 2011 through 2014, with 49% white, 27% Japanese, 12% Filipino, and 11% Native Hawaiian (Table 1). Comparisons between racial/ethnic groups found that a greater percentage of Native Hawaiians were younger (58.4% were aged 25–54 y), were never married (23.6%), reported annual income of less than $25,000 (30.3%), lived in rural areas (40.4%), had diabetes (14.7%), and were obese (45.5%) compared with whites. Filipino participants were also younger (57.6% were aged 25–54 y) and had a higher prevalence of diabetes than other participants. Nearly 94% of all participants reported that they have health insurance. Slightly fewer (89.8%) Native Hawaiians reported that they have health insurance. Native Hawaiians and Filipinos had the lowest percentages of dental visits, 61.3% and 69.3%, respectively, compared with whites and Japanese at 77.4% and 81.9%, respectively. All nonwhite groups had a higher percentage of excess tooth loss compared with whites. Native Hawaiians had the largest proportion (16.2%) of excess tooth loss, followed by Filipinos (13.1%), Japanese (11.6%), and whites (10.9%).

**Table 1:** Demographic Characteristics, by Race/Ethnicity, From the Hawai‘i Behavioral Risk Factor Surveillance System Population, 2011–2014

Characteristic	White (n = 6,569), %	Japanese (n = 3,641), %	Native Hawaiian (n = 1,506), %	Filipino (n = 1,655), %	Total (n = 13,371), %
**Sex**					
Female	49.5	55.5	53.0	54.7	52.8
Male	50.5	44.5	47.0	45.3	47.2
**Age group, y**					
25–54	43.8	31.1	58.4	57.6	44.1
55–64	24.6	23.3	18.3	20.8	22.8
≥65	31.6	45.6	23.3	21.6	33.2
**Education**					
High school diploma or less	29.9	29.8	58.7	49.0	36.7
Some college	33.0	35.3	26.3	34.7	33.3
College graduate	37.1	34.9	15.0	16.4	29.9
**Marital status**					
Never married	12.6	18.0	23.6	13.3	15.7
Married or partner	66.9	59.4	53.1	71.8	64.0
Divorced/separated	11.6	8.8	13.1	5.7	9.7
Widowed	8.9	13.8	10.3	9.2	10.7
**Annual income, $**					
≤24,999	17.2	13.6	30.3	26.6	19.3
25,000–49,999	22.2	28.1	28.0	35.2	27.2
50,000–74,999	18.2	19.4	16.3	14.3	17.6
≥75,000	42.5	38.9	25.4	23.9	35.9
**Insurance**					
Yes	94.0	96.4	89.8	91.6	93.8
No	6.0	3.6	10.2	8.4	6.2
**Metropolitan statistical area group**					
City center	29.5	42.9	25.1	28.6	33.1
Outside city center	25.3	36.2	34.5	38.3	32.4
Not in metropolitan statistical area	45.2	20.9	40.4	33.0	34.5
**Dental visit in past year**					
Yes	77.4	81.9	61.3	69.3	75.6
No	22.6	18.1	38.7	30.7	24.4
**Diabetes status**					
No diabetes	81.9	67.2	71.1	70.9	73.9
Prediabetes	10.9	19.1	14.1	14.4	14.6
Diabetes	7.2	13.8	14.7	14.8	11.6
**Body mass index (kg/m^2^)^a^**					
Normal weight (18.5–24.9)	41.7	48.3	21.2	41.4	41.5
Overweight (25.0–29.9)	36.4	36.5	33.2	36.5	36.1
Obese (≥30)	21.9	15.2	45.5	22.1	22.4

a Calculated as weight in kilograms divided by height in meters squared (kg/m^2^).

In the total study population (Table 2), excess tooth loss was significantly associated with age, education, income, and divorced/separated or widowed marital status. Presence of prediabetes or diabetes as well as not having had a dental visit were also significantly associated with excess tooth loss. The risk factors that were consistently associated with excess tooth loss across all 4 racial/ethnic groups were older age, low education, low income, and no dental visits (unadjusted). Using a stepwise analytical approach (Table 3) to develop a parsimonious model (inclusion of age, income, education, diabetes status, dental visit, and race/ethnicity), we found older age, lower income, and lower education as well as diagnosis of diabetes (but not prediabetes) and no dental visits in the past year were significantly associated with excess tooth loss, after adjusting for all variables in the regression model. Racial/ethnic differences compared with whites were no longer significant.

**Table 2:** Associations by Race/Ethnicity of Demographic and Health Characteristics With Having Lost 6 or More Teeth, Hawai‘i Behavioral Risk Factor Surveillance System Population, 2011–2014^a^

Characteristic	OR (95% CI)				
	White	Japanese	Native Hawaiian	Filipino	Total
**Sex**					
Female	1 [Reference]				
Male	1.3 (0.9–1.8)	1.4 (0.9–2.1)	0.7 (0.4–1.5)	0.8 (0.4–1.6)	1.1 (0.9–1.4)
**Age group, y**					
25–54	1 [Reference]				
55–64	3.7 (1.8–7.6)	2.0 (0.5–7.9)	3.0 (1.1–7.2)	2.4 (1.0–6.2)	2.6 (1.6–4.2)
≥65	8.7 (4.4–17.1)	8.1 (2.3–28.4)	6.8 (2.7–17.0)	4.0 (1.8–8.8)	5.5 (3.6–8.5)
**Education**					
High school diploma or less	4.5 (3.0–6.8)	6.2 (3.6–10.7)	3.4 (1.6–7.5)	3.3 (1.6–7.2)	4.8 (3.6–6.6)
Some college	1.9 (1.3–2.9)	2.5 (1.5–4.2)	1.9 (0.8–4.6)	1.3 (0.5–3.2)	2.0 (1.5–2.8)
College graduate	1 [Reference]				
**Marital status**					
Never married	1 [Reference]				
Married or partner	1.5 (0.8–2.8)	0.8 (0.4–1.6)	2.1 (0.9–5.1)	3.4 (1.1–10.6)	1.3 (0.8–2.0)
Divorced/separated	2.6 (1.3–5.2)	0.7 (0.3–1.8)	5.1 (1.5–17.7)	5.0 (1.2–20.4)	1.9 (1.1–3.3)
Widowed	4.9 (2.4–10.0)	1.4 (0.6–3.0)	18.7 (6.3–54.9)	7.5 (2.1–27.2)	3.5 (2.1–5.9)
**Annual income, $**					
≤24,999	6.5 (3.9–10.7)	7.1 (3.6–14.2)	4.4 (1.3–15.5)	4.7 (1.3–17.4)	6.0 (4.0–9.0)
25,000–49,999	4.1 (2.4–7.0)	3.3 (1.9–5.6)	2.5 (0.7–8.6)	2.7 (0.7–10.8)	3.3 (2.3–4.9)
50,000–74,999	1.8 (1.1–3.2)	2.4 (1.3–4.6)	1.7 (0.5–6.5)	2.3 (0.5–9.9)	2.2 (1.4–3.3)
≥75,000	1 [Reference]				
**Insurance**					
Yes	1 [Reference]				
No	0.5 (0.3–1.1)	1.2 (0.2–8.1)	0.3 (0.1–1.3)	0.5 (0.2–1.5)	0.6 (0.3–1.5)
**Metropolitan statistical area group**					
City center	1 [Reference]				
Outside city center	1.4 (0.8–2.4)	0.8 (0.5–1.5)	0.9 (0.3–2.5)	1.1 (0.4–3.0)	1.0 (0.7–1.5)
Not in metropolitan statistical area	1.6 (1.1–2.3)	1.1 (0.7–1.6)	0.8 (0.4–1.6)	1.2 (0.6–2.4)	1.1 (0.9–1.5)
**Dental visit in past year**					
Yes	1 [Reference]				
No	3.1 (2.2–4.5)	2.0 (1.3–3.2)	2.9 (1.5–5.7)	2.2 (1.1–4.2)	2.5 (1.9–3.2)
**Diabetes status**					
No diabetes	1 [Reference]				
Prediabetes	2.1 (1.3–3.4)	1.0 (0.6–1.7)	2.9 (1.1–7.6)	1.1 (0.5–2.6)	1.5 (1.1–2.0)
Diabetes	4.2 (2.5–7.3)	1.4 (0.8–2.3)	3.9 (1.7–8.9)	2.5 (1.1–5.6)	2.4 (1.7–3.3)
**Body mass index (kg/m^2^)^b^**					
Normal weight (18.5–4.9)	1 [Reference]				
Overweight (25.0–29.9)	0.9 (0.6–1.3)	1.1 (0.7–1.7)	0.5 (0.2–1.3)	0.7 (0.4–1.1)	0.9 (0.6–1.2)
Obese (≥30)	1.7 (1.1–2.7)	0.9 (0.5–1.7)	0.5 (0.2–1.2)	0.5 (0.2–1.6)	0.9 (0.7–1.3)

Abbreviations: CI, confidence interval; OR, odds ratio.

a Analyses are unadjusted except for the total population, whose analysis was adjusted for ethnicity.

b Calculated as weight in kilograms divided by height in meters squared (kg/m^2^).

**Table 3:** Stepwise Multivariable Models for Excessive Tooth Loss (6 or More Teeth) in Adults, Hawai‘i Behavioral Risk Factor Surveillance System Population, 2011–2014

Characteristic	Odds Ratio (95% Confidence Interval)	*P* Value^a^
**Age group, y**		
25–54	1 [Reference]	
55–64	2.58 (1.57–4.25)	<.001
≥65	5.47 (3.41–8.79)	<.001
**Annual income, $**		
≤24,999	2.42 (1.49–3.92)	<.001
25,000–49,999	1.83 (1.20–2.78)	.005
50,000–74,999	1.51 (0.97–2.35)	.07
≥75,000	1 [Reference]	
**Education**		
High school diploma or less	2.62 (1.88–3.66)	<.001
Some college	1.51 (1.07–2.11)	.02
College graduate	1 [Reference]	
**Diabetes status**		
No diabetes	1 [Reference]	
Prediabetes	1.15 (0.80–1.65)	.46
Diabetes	1.64 (1.15–2.35)	.007
**Dental visit in past year**		
Yes	1 [Reference]	
No	2.29 (1.67–3.13)	<.001
**Race/ethnicity**		
White	1 [Reference]	
Japanese	1.24 (0.90–1.72)	.19
Native Hawaiian	1.39 (0.92–2.12)	.12
Filipino	1.34 (0.86–2.06)	.19

a Determined by Wald tests.

## Discussion

This study provides confirmatory and new data about tooth loss in Hawai‘i. First, the data confirm the association between tooth loss and demographic and health risk factors among multiple racial/ethnic populations in Hawai‘i, one of the most demographically diverse states. The demographic factors associated with excess tooth loss (older age, lower education attainment, and lower income) are consistent with findings in the existing literature (2,12,13). Presence of diabetes (16–19) and lack of a dental visit in the past year (9,14,15) were also significantly associated with excess tooth loss, despite more than 90% of the participants having health insurance coverage. Second, before accounting for demographic and health risk factors, Native Hawaiians had the highest prevalence of tooth loss among the racial/ethnic groups examined.

More specifically, we found that the association between loss of 6 or more teeth and race/ethnicity was not significant after adjusting for demographic variables, diabetes status, and dental visits. Public health policy and programs aimed at improving low income and education, reducing diabetes prevalence, and increasing dental visits are likely to benefit all racial/ethnic groups. The American Dental Association reports that the number one reason for not visiting a dentist more frequently (among those without a visit in the last 12 months) is cost (59%), followed by “afraid of dentist” (22%), and “inconvenient location or time” (19%) (21). If indeed access to dental care is a barrier to preventive dental services, then public health officials may want to consider the possibility of health policies to improve affordability and access at convenient locations to the highest risk populations (eg, low income, older age).

Strengths of this study include a large sample size (>13,000) of a diverse, multiethnic population. However, the proportion of Native Hawaiians and Filipinos was less than 25% of the study sample. Limitations of this study are the use of cross-sectional data and the recall bias of participants inherent in this type of study design.

An unexpected finding of this study was lack of association between excess tooth loss and BMI among Japanese, Native Hawaiians, and Filipino adults. Studies have suggested increased risk for tooth loss among obese people (22,23) and different “healthy weight” cut-offs for different racial/ethnic groups (24). We used the same “healthy weight” cut-off across all racial/ethnic groups.

Hawai‘i received an “F” on 4 sequential (2010, 2011, 2012, and 2014) Pew Trusts reports on oral health, which assessed 50 states (25–28). In the 2010 and 2011 reports, 8 policy benchmarks were used (25,26): 1) Share of high-risk schools with sealant programs; 2) Hygienists can place sealants without dentist’s prior exam; 3) Share of residents on fluoridated community water supplies; 4) Share of Medicaid-enrolled children getting dental care; 5) Share of dentists’ median retail fees reimbursed by Medicaid; 6) Pays medical providers for early preventive dental health care; 7) Authorizes new primary care dental providers; and 8) Tracks data on children’s dental health. In the 2010 report, Hawai‘i met 2 of the 8 benchmarks: 2 and 4. In the 2011 report, Hawai‘i met only 1 of the 8 benchmarks: benchmark 4. In 2013, the state of Hawai‘i obtained funding through a Centers for Disease Control and Prevention cooperative agreement to build basic oral health capacity. In 2015, the Hawai‘i State Department of Health conducted an oral health survey among third-grade children (29). The survey report found that “In Hawaii, low-income, Micronesian, Native Hawaiian, Other Pacific Islander and Filipino children have poorer oral health outcomes [than white and Japanese children],” and said “The findings presented in this report support the need for *culturally appropriate community-based prevention programs, screening and referral services, and restorative dental care to improve the oral health of Hawaii’s children.*” Though oral health disparities have been identified and some isolated community-based prevention programs exist, actions should be taken to address oral health disparities systematically.

Before adjusting for socioeconomics, diabetes, and dental visits, all nonwhite groups had a higher percentage of excess tooth loss compared with whites. Native Hawaiians had the largest portion of excess tooth loss. After adjusting for socioeconomics, diabetes, and dental visits, the associations disappeared in all groups. This suggests there may be common programs and health policies that improve oral health equity for all low-socioeconomic populations and diabetes patients to prevent excess tooth loss (eg, community-based care).

The ethnic/racial differences in strength of associations between tooth loss and demographic and socioeconomic variables, diabetes status, and dental visits found in the unadjusted model may be due to the distinct culture that each ethnic group possesses. (Race is associated with biology, whereas ethnicity is associated with culture.) Culture refers to the cumulative deposit of knowledge, experience, beliefs, values, attitudes, meanings, hierarchies, religion, notions of time, roles, spatial relations, concepts of the universe, and material objects and possessions acquired by a group of people in the course of generations through individual and group striving (30). Therefore, culturally appropriate tooth loss prevention needs to include both generic components for all racial/ethnic populations and be customized for specific populations. Further, this cultural diversity suggests that future health equity studies should simultaneously focus on increasing our cultural understanding.

This study filled a research gap by examining individual contributions of known factors among 4 different racial/ethnic groups. It found that the strength of associations between the loss of 6 or more teeth and demographic variables, diabetes status, and dental visits varied among white, Japanese, Filipino, and Native Hawaiian participants. It found that the association between the loss of 6 or more teeth and race/ethnicity was not significant after adjusting for demographic variables, diabetes status, and dental visits. It also revealed health inequity among the 4 ethnic groups; a greater proportion of Native Hawaiians, indigenous people of Hawai‘i, had loss of 6 or more teeth than the other compared ethnic groups in Hawai‘i. The findings suggest a need for programs and policies that would improve access to oral health care in Hawai‘i for residents with low levels of income and education, those with low dental care utilization, and those with diabetes.

## Post-Test Information

To obtain credit, you should first read the journal article. After reading the article, you should be able to answer the following, related, multiple-choice questions. To complete the questions (with a minimum 75% passing score) and earn continuing medical education (CME) credit, please go to http://www.medscape.org/journal/pcd. Credit cannot be obtained for tests completed on paper, although you may use the worksheet below to keep a record of your answers.

You must be a registered user on http://www.medscape.org. If you are not registered on http://www.medscape.org, please click on the “Register” link on the right hand side of the website.

Only one answer is correct for each question. Once you successfully answer all post-test questions, you will be able to view and/or print your certificate. For questions regarding this activity, contact the accredited provider, CME@medscape.net. For technical assistance, contact CME@medscape.net. American Medical Association’s Physician’s Recognition Award (AMA PRA) credits are accepted in the US as evidence of participation in CME activities. For further information on this award, please go to https://www.ama-assn.org. The AMA has determined that physicians not licensed in the US who participate in this CME activity are eligible for *AMA PRA Category 1 Credits™*. Through agreements that the AMA has made with agencies in some countries, AMA PRA credit may be acceptable as evidence of participation in CME activities. If you are not licensed in the US, please complete the questions online, print the AMA PRA CME credit certificate, and present it to your national medical association for review.

## Post-Test Questions

### Study Title: Preventable Tooth Loss in Hawai‘i: The Role of Socioeconomic Status, Diabetes, and Dental Visits

#### CME Questions

1. You are advising a health maintenance organization in Hawaii on tooth loss prevention. According to the analysis of data from the Hawaii Behavioral Risk Factor Surveillance Survey by Deguchi and colleagues, which of the following statements about the associations of tooth loss in Hawaii with race or ethnicity and other demographic factors is ***correct***?
  - Japanese people had the largest proportion of excess tooth loss
  - In the total study population, excess tooth loss was significantly associated with older age; low educational level; low income; and divorced, separated, or widowed marital status
  - Demographic factors associated with excess tooth loss differed considerably from those found in previous studies
  - Older age was associated with excess tooth loss only among Filipinos
2. According to the analysis of data from the Hawaii Behavioral Risk Factor Surveillance Survey by Deguchi and colleagues, which of the following statements about associations of tooth loss in Hawaii with various health and healthcare factors is ***correct***?
  - After adjustment for demographics, socioeconomics, diabetes status, and dental visits, associations between tooth loss and race or ethnicity were not significant
  - In the total study population, excess tooth loss was not significantly associated with presence of diabetes
  - In a step-wise analytical approach adjusting for all variables in the regression model, having no previous dental visit was not significantly associated with excess tooth loss
  - Excess tooth loss was significantly associated with body mass index (BMI) among Japanese, Native Hawaiians, and Filipino adults
3. According to the analysis of data from the Hawaii Behavioral Risk Factor Surveillance Survey by Deguchi and colleagues, which of the following statements about the clinical implications of factors associated with tooth loss in Hawaii is ***correct***?
  - Public health policy and programs aimed to improve diabetes status and dental visits are likely to benefit only Filipinos
  - The American Dental Association reports that the leading reason for not visiting a dentist more frequently is fear of the dentist
  - Public health officials should consider health policies to improve dental care affordability and access to highest-risk populations (low income, older age, diabetes, etc.)
  - Pew Trusts Reports on oral health showed continued improvement in quality of care in Hawaii from 2010 to 2014
